# Tumefactive Multiple Sclerosis of the Cervical Spinal Cord: A Rare Case Report

**DOI:** 10.7759/cureus.6754

**Published:** 2020-01-23

**Authors:** Ahmed Mamilly, Asala Aslan, Nimer Adeeb, Aya Al Asfari, Hugo Cuellar

**Affiliations:** 1 Radiology, Louisiana State University, Shreveport, USA; 2 Neurosurgery, Louisiana State University, Shreveport, USA; 3 Neurosurgery, Louisiana State University Health Sciences Center Shreveport, Shreveport, USA

**Keywords:** spinal cord, cervical, multiple sclerosis, tumefactive

## Abstract

Tumefactive multiple sclerosis (TMS) is a rare variant of multiple sclerosis (MS) with atypical features that pose a diagnostic challenge. In this study, we report a case of cervical MS in a 19-year-old patient that was diagnosed based on the MRI findings and cerebrospinal fluid analysis. The patient was treated with high-dose steroid and five sessions of plasma exchange with significant improvement.

## Introduction

Tumefactive multiple sclerosis (TMS) is one of the rare variants of multiple sclerosis (MS) with atypical features that pose a diagnostic challenge since it is difficult to distinguish from a true central nervous system neoplasm. Spinal cord TMS is extremely rare and was only documented few times in the literature [1,2]. In this study, we report the first documented case of cervical TMS in a young patient.

## Case presentation

A 19-year-old African-American female presented to the emergency department for evaluation of subacute onset of left-sided hemianesthesia that started in the left hand and gradually progressed proximally to the entire left upper and lower extremities, and then the right side of the body over a course of two weeks. The patient reported associated weakness and bifrontal throbbing headache. She also reported decreased bowel and bladder sensation, but no episodes of bowel or bladder incontinence.

On physical examination, the patient had 4/5 strength in the left upper extremity and 5/5 strength in the right upper extremity. She had decreased sensation to light touch on the left side of her body, and a mild decrease in rectal tone. 

**Figure 1:**
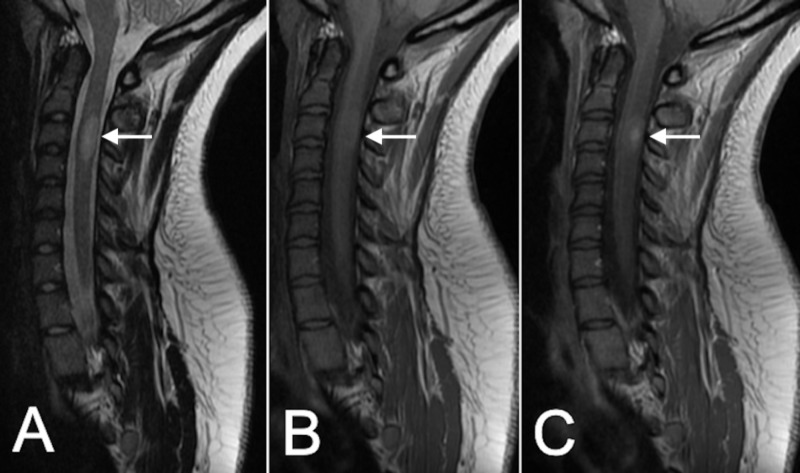
Initial MRI Presentation MRI shows a C3 cervical intramedullary lesion (arrow) that is hyperintense on T2-weighted images (A), isointense on T1-weighted images (B), with areas of contrast enhancement (C).

MRI of brain and spine showed sharply delineated ovoid mass within the spinal cord at the level of C3 that measured approximately 1.7 cm (SI) x 6.4 mm (AP). This lesion had high intensity on T2 and was isointense to the spinal cord on T1, with partial central enhancement. No syringomyelia or surrounding cord edema was identified, but the cord was expanded at the level of the mass (Figure 1).

Based on the radiological findings, low-grade astrocytoma, ependymoma, and inflammatory or demyelinating lesion were in the differential diagnosis. 

The patient was then started on steroids and cerebrospinal fluid (CSF) was obtained. CSF analysis was positive for oligoclonal bands. This raised the suspicion for a demyelinating lesion, and the patient was treated with combination of high-dose steroid and five plasma exchange sessions. 

**Figure 2:**
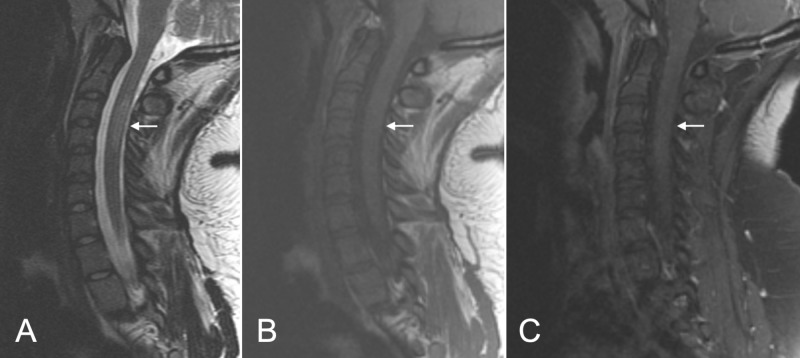
Follow-up MRI Follow-up MRI at two months shows mild reduction of the lesion size and intensity (arrow) on T2-weighted images (A) and T1-weighted images (B), with no signs of contrast enhancement (C).

After completing the treatment inpatient, the patient started to have significant improvement in her left side weakness and numbness. She was cleared by physical and occupational therapy to be discharged home. At two-month follow-up, the patient continued to do well, and the repeat MRI showed mild reduction in the size of the lesion and no contrast enhancement, indicating an inactive inflammatory lesion (Figure 2). 

## Discussion

TMS is a rare variant of classical MS, where in the criterion of having two or more lesions separated by time and space is not met. The prevalence of TMS is estimated to be 1-3/1,000 cases of MS with an annual incidence of 0.3/100,000 [3]. It often characterized by a solitary lesion sized > 2 cm, with mass effect, edema, and ring enhancement on MRI [3-5]. Due to its rarity and unique characteristic, TMS usually mimics tumors, infections, vascular lesions, and inflammatory lesions, and the diagnosis can be challenging. Diagnosis is usually confirmed by biopsy, which shows identical histopathological features to MS: infiltrating foamy macrophages, reactive gliosis, myelin loss, and relative axonal preservation [4].

In this study, we reported a case of cervical TMS in a 19-year-old patient. To our knowledge, this is the third case in the literature to report spinal TMS, and the first to show a cervical lesion [1,2]. Due to the unusual location, the initial diagnostic effort is to rule out spinal intramedullary tumors. Differentiation between the two is more challenging than their brain counterpart. This is partly due to the relatively smaller lesions, and the limited use of perfusion-weighted MRI and spectroscopy. Yaghi et al. reported a case of spinal cord TMS that was misdiagnosed as a tumor, and the patient underwent complete surgical resection only to find that the lesion was in fact a TMS [1]. Therefore, having high suspicion in addition to CSF analysis is usually helpful following the radiological findings. Despite being the gold standard diagnostic tool, biopsy can be very risky in high spinal cord lesions. Similar to TMS in the brain, the lesion was being treated with high-dose steroid and plasma exchange. On follow-up MRI, the lesion had mildly decreased in size with no signs of active inflammation. 

## Conclusions

In this study we report a case of cervical MS in a 19-year-old patient that was diagnosed based on the MRI findings and CSF analysis. The patient was treated with high-dose steroid and five sessions of plasma exchange with significant improvement. 
